# First detection and genetic characterization of ungulate tetraparvovirus 2 and ungulate tetraparvovirus 4 in special livestock on the Qinghai-Tibet Plateau in China

**DOI:** 10.1186/s12985-019-1167-z

**Published:** 2019-05-02

**Authors:** Yangyang Pan, Yun Wang, Meng Wang, Qian Zhang, Abdul Rasheed Baloch, Jun Zhou, Jing Ma, Jam Kashif, Gengquan Xu, Libin Wang, Jiangfeng Fan, Yan Cui, Sijiu Yu

**Affiliations:** 1 Gansu Agricultural University, College of Veterinary Medicine, Lanzhou, 730070 China; 2 Anning Branch Lanzhou Genegal Hospital, Lanzhou, 730070 China; 3 University of South Bohemia in České Budějovice, Faculty of Fisheries and Protection of Waters, South Bohemian Research Center of Aquaculture and Biodiversity of Hydrocenoses, Research Institute of Fish Culture and Hydrobiology, Zátiší 728/II, 389 25 Vodňany, Czech Republic; 4 Animal Health and Epidemiology Center in Chengguang District, Lanzhou, 73000 China; 5 Department of Veterinary Medicine, Sindh Agriculture University Tandojam, Tandojam, Pakistan

**Keywords:** Ungulate tetraparvovirus 2, Ungulate tetraparvovirus 4, Tibetan pig, Tibetan sheep, Qinghai-Tibet plateau

## Abstract

*Tetraparvovirus*, formerly known as *Partetravirus*, is a newly discovered genus in the family *Parvoviridae* that is considered phylogenetically distinct from other parvoviruses. However, nothing is known about the prevalence of *Tetraparvovirus* in special livestock living on the Qinghai-Tibet Plateau of China, such as Tibetan pigs and Tibetan sheep. A pair of special primers was designed based on the conserved regions in the genome of ungulate tetraparvovirus 2 (P-PARV4) and ungulate tetraparvovirus 4 (O-PARV4) and was used to detect P-PARV4 in domestic pigs and Tibetan pigs and O-PARV4 in ovines and Tibetan sheep. The results showed a 15.59 and 9.38% prevalence of P-PARV4 in domestic pigs (18.96% in Gansu Province and 11.76% in Qinghai Province) and Tibetan pigs (14.28% in Gansu Province and 4.44% in Qinghai Province), respectively, and a 7.31 and 5.20% prevalence of O-PARV4 in ovines (6.61% in Gansu Province and 8.00% in Qinghai Province) and Tibetan sheep (4.55% in Gansu Province and 5.50% in Qinghai Province), respectively. The prevalence of P-PARV4 was 14.76% (31/210) for ≤1-month-old pigs and 10.58% (20/189) for > 1-month-old pigs, and the positive rates of O-PARV4 were 7.65% (18/235) for ≤1-month-old sheep and 5.05% (11/218) for > 1-month-old sheep. The phylogenetic analysis of NS1, VP1, VP2 and the whole PARV4-related provirus genome demonstrated that both P-PARV4 and O-PARV4 sequences in this study were more closely related to the sequences of other strains discovered in the same genus of animals. The identity analyses for the full-length VP2 genomes of O-PARV4 revealed 98.84–100.00% sequence identity among the 7 strains and the previously reported strain, which was 98.60–99.28% for P-PARV4. In the present study, for the first time, we have provided comprehensive information regarding the widespread infection of P-PARV4 and O-PARV4 in special livestock on the Qinghai-Tibet Plateau in China. Our present findings highlight the importance of epidemiologic surveillance to limit the spread of *Tetraparvovirus* in livestock at high altitudes in China.

**Electronic supplementary material:**

The online version of this article (10.1186/s12985-019-1167-z) contains supplementary material, which is available to authorized users.

Members of the family *Parvoviridae* are small, non-enveloped viruses with single-stranded DNA genomes of approximately 5 kilobases (kb) [1]. The genome of most parvoviruses contains two open reading frames (ORFs) separated by a small non-coding region, where ORF1 encodes non-structural proteins (NS) and ORF2 encodes two overlapping viral capsid proteins (VP1/VP2) [1]. The development of molecular biology-based detection methods led to the discovery that *parvoviruses* are widespread pathogens that cause a wide range of diseases in livestock and humans [2]. According to the International Committee on the Taxonomy of Viruses (ICTV) 2018, *Tetraparvovirus* (previously proposed as “Partetravirus”) is a newly established genus in the *Parvoviridae* family that includes primate tetraparvovirus 1 (formerly known as human parvovirus 4, PARV-4) and six novel PARV4-related animal parvoviruses described in the past decade, viz.*,* chiropteran tetraparvovirus 1, primate tetraparvovirus 1, ungulate tetraparvovirus 1 (bovine hokovirus 1, B-PARV4), ungulate tetraparvovirus 2 (porcine hokovirus, P-PARV4), ungulate tetraparvovirus 3 (porcine Cn virus, CnP-PARV4) and ungulate tetraparvovirus 4 (ovine hokovirus, O-PARV4) [3]. The prevalence of P-PARV4 and B-PARV4 has been widely reported throughout the world, including Gansu and Qinghai Provinces in China [3, 4]. We also previously discovered B-PARV4 in domestic yaks (*Bos grunniens*) [3], which are also considered special livestock on the Qinghai-Tibet Plateau. However, only one report describes the members of the O-PARV4 species that was discovered in Hong Kong in 2011 [5]. To date, no other report of this novel parvovirus exists anywhere in the world. Here, for the first time, we report P-PARV4 and O-PARV4 infections in livestock from the Qinghai-Tibet Plateau in China.

The Qinghai-Tibet Plateau is an extensive pastoral and semi-pastoral area located in Asia, northeast of the Himalayas. It is the highest region on earth (altitudes > 3000 m, average annual temperature < 0 °C) and includes the Qinghai, Gansu Sichuan and Tibet Provinces in northwestern China. Yak, Tibetan pigs and Tibetan sheep are the main livestock that live on the Qinghai-Tibet Plateau in China. Because they live in a cold and harsh environment on a plateau for an extended period of time, the livestock in these areas have undergone specific selection to enrich disease resistance-related gene categories in their genome [6, 7]. However, some pathogenic infections have been discovered in these livestock in recent years, such as hepatitis E virus (HEV) [8], *Brucella spp.* [9], bovine viral diarrhoea virus (BVDV) [10] and B-PARV4 [3] infections in yaks, swine influenza A virus (SIV) [11] and highly pathogenic porcine reproductive and respiratory syndrome virus (HP-PRRSV) [12] infections in Tibetan pigs, and the infection rates of these pathogens were lower than those in regular production animals [9–11]. All the data indicate that livestock on the Qinghai-Tibet Plateau in China are susceptible to pathogenic infections. P-PARV4 and O-PARV4 infections in China have been reported for many years. Additionally, the prevalence of B-PARV4 in yaks that were farmed together with Tibetan pigs and Tibetan sheep, which we have been involved in research on P-PARV4 and B-PARV4 and the surveillance of livestock infections on the Qinghai-Tibet Plateau. Thus, it is necessary to investigate and prevent P-PARV4 and O-PARV4 infections in the highland region in China.

Our present study aimed to investigate the distribution of P-PARV4 and O-PARV4 in domestic pigs, Tibetan pigs, ovines and Tibetan sheep on the Qinghai-Tibet Plateau in China based on the amplification of tetraparvovirus VP2 by polymerase chain reaction (PCR) using primers we designed based on the conserved regions in the VP2 sequences of P-PARV4 and O-PARV4. Full-length sequences of NS1, VP1, and VP2 and nearly full-length sequences of the identified PARV4-related viruses (seven strains from pigs, three from sheep) were also determined and analysed. The results should provide a solid foundation for the improved control of *Tetraparvovirus* infection in livestock in this region of China.

## Methods

From May 2016 to June 2017, blood samples from 218 domestic pigs, 181 Tibetan pigs, 246 ovines and 219 Tibetan sheep were collected from Gansu and Qinghai Provinces surrounding the Qinghai-Tibet Plateau in China with the assistance of the Animal Health and Epidemiology Center and Veterinary Department (see Additional file 1). The samples were collected from apparently healthy animals. The specimens were grouped according to their species, ages and geographical region (see Additional file 1). Genomic DNA was extracted from 100 μL of whole blood from each sample using the E-Z 96® Blood DNA Kit (Omega, Norcross, GA, United States) according to the manufacturer’s instructions. The extracted DNA was stored at − 20 °C until required for PARV4-related detection and PCR amplification of full-length sequences.

To detect PARV4-related viruses in special livestock by PCR, one pair of special primers, PARV4-F and PARV4-R, was designed (see Additional file 2) using Primer Premier 6.24, targeting a 640-bp fragment of the conserved VP2 region based on multiple alignment of the reference P-PARV4 (EU200676) and O-PARV4 (JF504700) genomic sequences available in the National Center for Biotechnology Information (NCBI) GenBank database. PCR products were confirmed by 1.2% agarose gel stained with ethidium bromide and were visualized under ultraviolet light. DNA of P-PARV4 in domestic pigs from eastern China and sterilized water were used as the PCR positive and negative controls, respectively.

Seven P-PARV4-positive samples and eight O-PARV4-positive samples were randomly selected based on region and species. Each of the 4 pairs of primers that produced overlapping PCR products was designed based on the reference P-PARV4 (EU200676) and O-PARV4 genomes (JF504700) (see Additional file 2), which were used to obtain the PARV4-related genome consisting of all the ORFs. The four different PARV4-related provirus genome PCR amplicons from each individual were purified using the QIAquick Gel Extraction Kit (Qiagen, Germany), cloned utilizing a TA cloning kit (TaKaRa TA Kit; Dalian, China) and subsequently transformed into competent *Escherichia coli* cells (DH5α). Four purified recombinant plasmids were sequenced (Sangon Biotech, Shanghai, China), assembled, and edited by MEGA 7.1 [13] and DNAMAN 9.0 software to produce the final sequences of the viral genomes. Seven complete genomic sequences for P-PARV4 (MG365914-MG365920) and eight sequences for O-PARV4 (MG365906-MG365913) were deposited in the GenBank database and used for further analysis. Full-length NS1, VP1, and VP2 sequences and nearly full-length sequences of seven P-PARV4 strains and eight O-PARV4 strains detected in this study were independently used in sequence alignment and phylogenetic and identity analyses with the B-PARV4, O-PARV4, and P-PARV4 sequences available in the NCBI GenBank database.

## Results

The PARV4-related viruses were detected from 864 livestock blood samples by PCR targeting VP2 regions. For P-PARV4, the blood samples of domestic pigs and Tibetan pigs at two different ages were collected from Gansu and Qinghai Provinces. Positive samples for P-PARV4 in domestic pigs were found in 22 of 116 pigs (18.97%) in Gansu Province and 12 of 102 pigs (11.76%) in Qinghai Province. In Gansu Province, the positive rates of P-PARV4 in domestic pigs were 20.31% (13/64) for ≤1-month-old pigs and 17.31% (9/52) for > 1-month-old pigs and were 13.56% (8/59) for ≤1-month-old pigs and 9.30% (4/43) for > 1-month-old pigs in Qinghai Province (see Additional file 1). In Tibetan pigs, the positive rates of P-PARV4 in Gansu Province were 14.29% (13/91), including 16.33% (8/49) for ≤1-month-old pigs and 11.90% (5/42) for > 1-month-old pigs, and were 4.44% (4/90) in Qinghai Province, including 5.26% (2/38) for ≤1-month-old pigs and 3.85% (2/52) for > 1-month-old pigs.

For O-PARV4, positive samples in Gansu Province were found in 8 of 121 ovines (6.61%) and 5 of 110 (4.55%) Tibetan sheep; additionally, the positive rates of P-PARV4 in ovines were 7.46% (5/67) for ≤1-month-old ovines and 5.56% (3/54) for > 1-month-old ovines, and they were 6.45% (4/62) for ≤1-month-old ovines and 2.08% (1/48) for > 1-month-old ovines (see Additional file 1). In Qinghai Province, positive samples for O-PARV4 were found in 10 of 125 ovines (8.00%) and 6 of 109 Tibetan sheep (5.50%). The positive rates of O-PARV4 in ovines were 8.33% (6/72) for ≤1-month-old ovines and 7.55% (4/53) for > 1-month-old ovines, and they were 6.67% (3/45) for ≤1-month-old ovines and 4.76% (3/63) for > 1-month-old ovines (see Additional file 1).

**Fig. 1:**
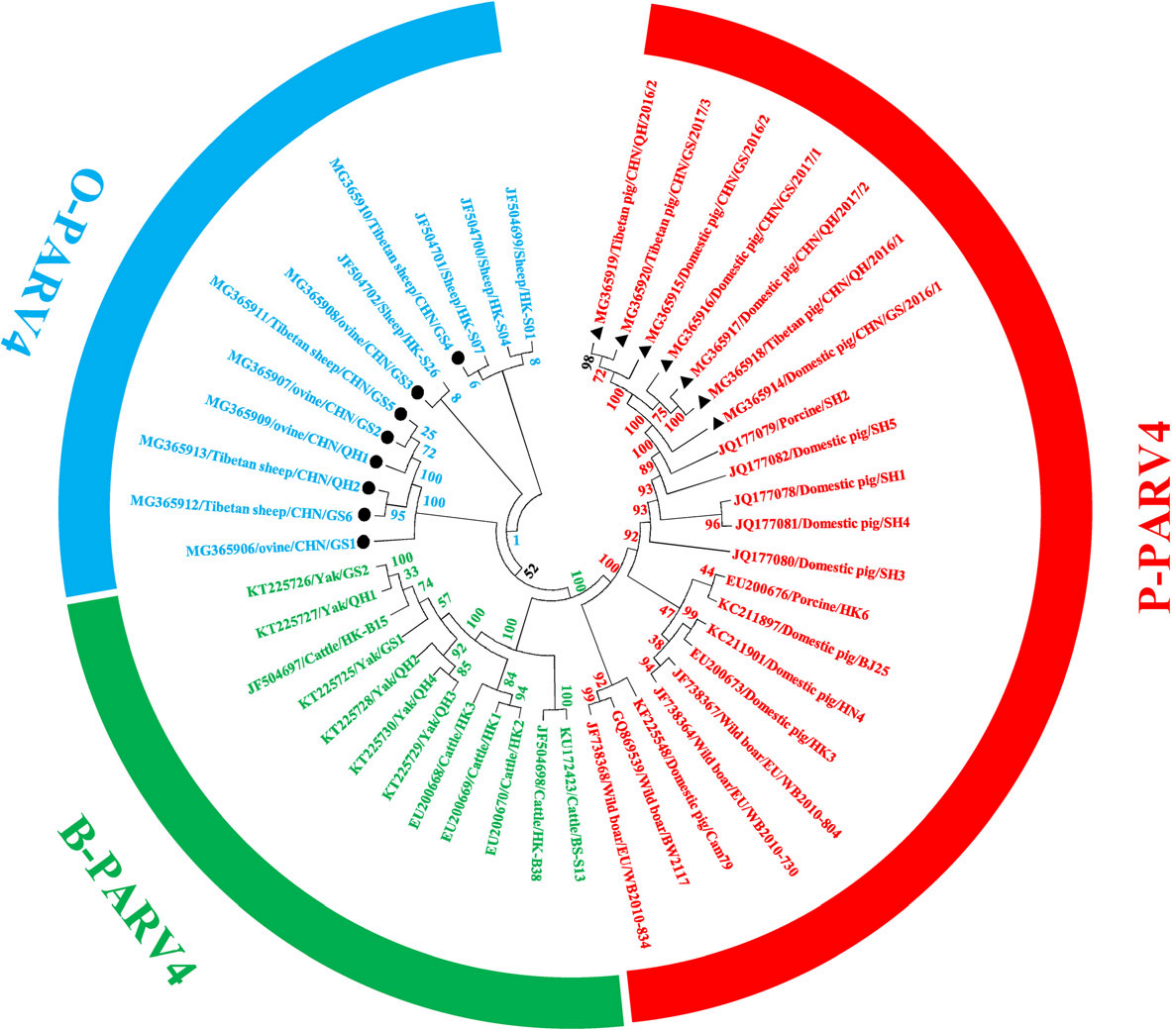
Maximum likelihood (ML) phylogenetic tree constructed from complete PARV4-related genomic sequences**.** The ML phylogenetic tree was constructed using the complete PARV4-related genomic sequences from the seven P-PARV4 strains and eight O-PARV4 strains detected in this study (submitted to the GenBank nucleotide sequence database with accession numbers MG368906-MG368920) and 30 reference sequences obtained from the GenBank nucleotide sequence database. One thousand replications were performed to calculate bootstrap values (indicated on the tree). The strains identified in the phylogenetic tree are indicated by GenBank accession numbers and breeds and strains. The seven newly identified P-PARV4 strains and eight O-PARV4 strains described in the present study are indicated by “▲” and “●”, respectively. P-PARV4, O-PARV4 and B-PARV4 strains are shown in red, blue and green around the circumference of the figure, respectively

**Fig. 2:**
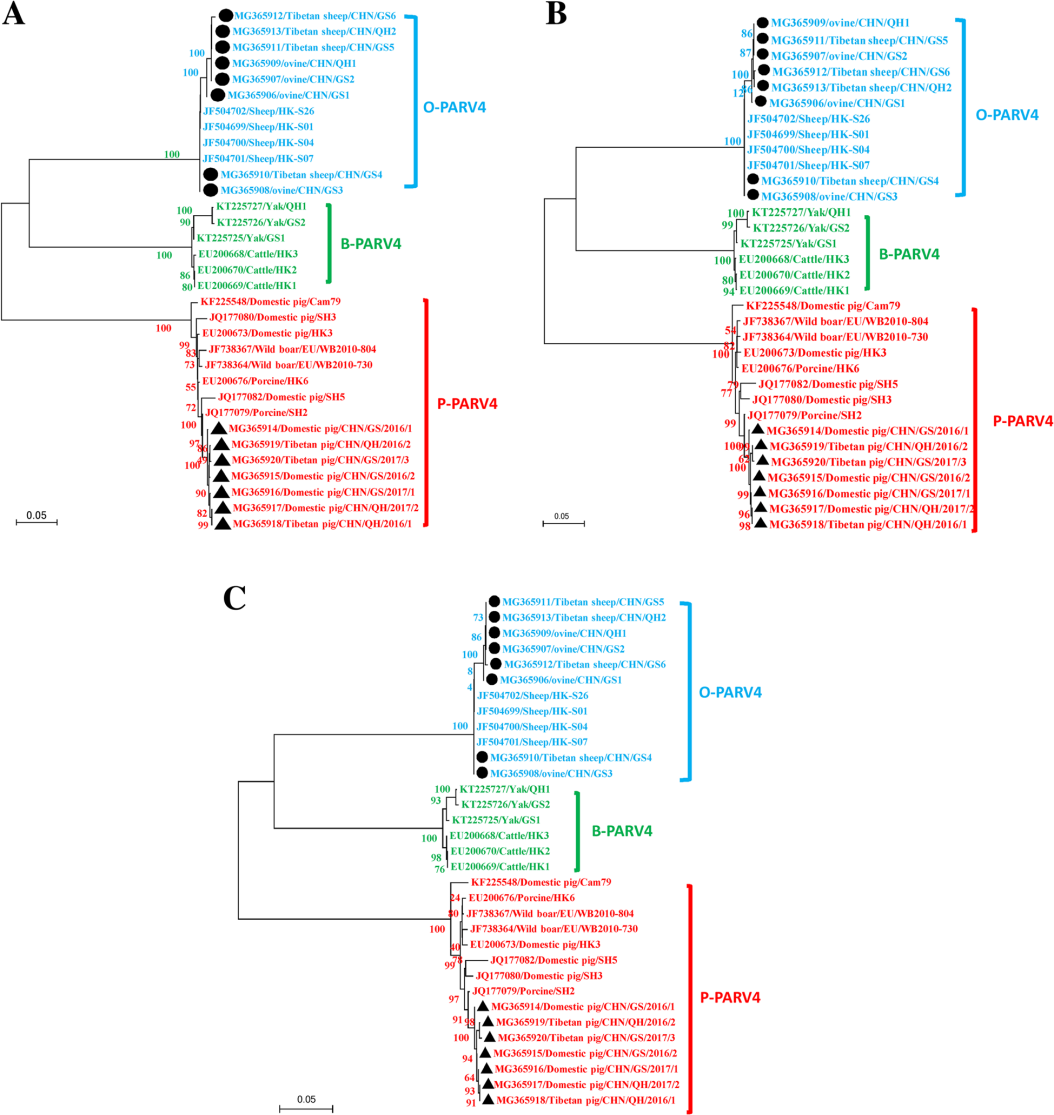
Phylogenetic analysis of PARV4-related viruses based on NS1(A), VP1(B) and VP2(C). The phylogenetic tree was constructed by using maximum likelihood (ML) with MEGA 7.01 software (http://www.megasoftware.net). Bootstrap values were calculated with 1000 replicates. The number on each branch indicates bootstrap values. The strains identified in the phylogenetic tree are indicated by GenBank accession numbers and breeds and strains. The seven P-PARV4 strains and eight O-PARV4 strains newly identified in the present study are indicated by “▲” and “●”, respectively. P-PARV4, O-PARV4 and B-PARV4 are shown in red, blue and green, respectively. Scale bar indicates nucleotide substitutions per site

The phylogenetic trees based on the complete genome sequence and the NS1, VP1, and VP2 genes clearly showed three main branches, encompassing B-PARV4, O-PARV4, and P-PARV4. The seven new P-PARV4 genomes in domestic pigs and Tibetan pigs clustered on the same branch as all known P-PARV4 genomes. Additionally, the eight new O-PARV4 genomes in ovines and Tibetan sheep clustered on the same branch as all known O-PARV4 genomes (Figs. 1 and 2). On the phylogenetic tree based on the complete genome sequence of PARV4-related provirus (Fig. 1), the main branch of P-PARV4 was divided into three additional branches, and the seven new P-PARV4 strains in domestic pigs and Tibetan pigs were closely related to each other and formed one branch with the P-PARV4 strains isolated from domestic pigs in eastern China (JQ177078-JQ177082) (Fig. 1). The main branch of O-PARV4 strains was also divided into three further branches; 6 of the 8 new O-PARV4 strains in ovines and Tibetan sheep were closely related to each other and formed a separate branch. However, the new O-PARV4 strains of MG365908/ovine/CHN/GS3 discovered in this study formed one branch with JF5004702/Sheep/HK-S26, and MG365910/Tibetan sheep/CHN/GS4 formed one branch with JF5004699/Sheep/HK-S01, JF5004700/Sheep/HK-S04 and JF5004701/Sheep/HK-S07 that was isolated from sheep in Hong Kong, China (Fig. 1). The phylogenetic tree of the NS1, VP1 and VP2 genes indicated that MG365908/ovine/CHN/GS3 and MG365910/Tibetan sheep/CHN/GS4 were also located in the same separate subcluster (Fig. 2). The identity analyses for full-length VP2 genomes of O-PARV4 revealed 98.84–100.00% sequence identity among the 7 strains and the previously reported strain, which for P-PARV4 was 98.60–99.28%.

**Fig. 3:**
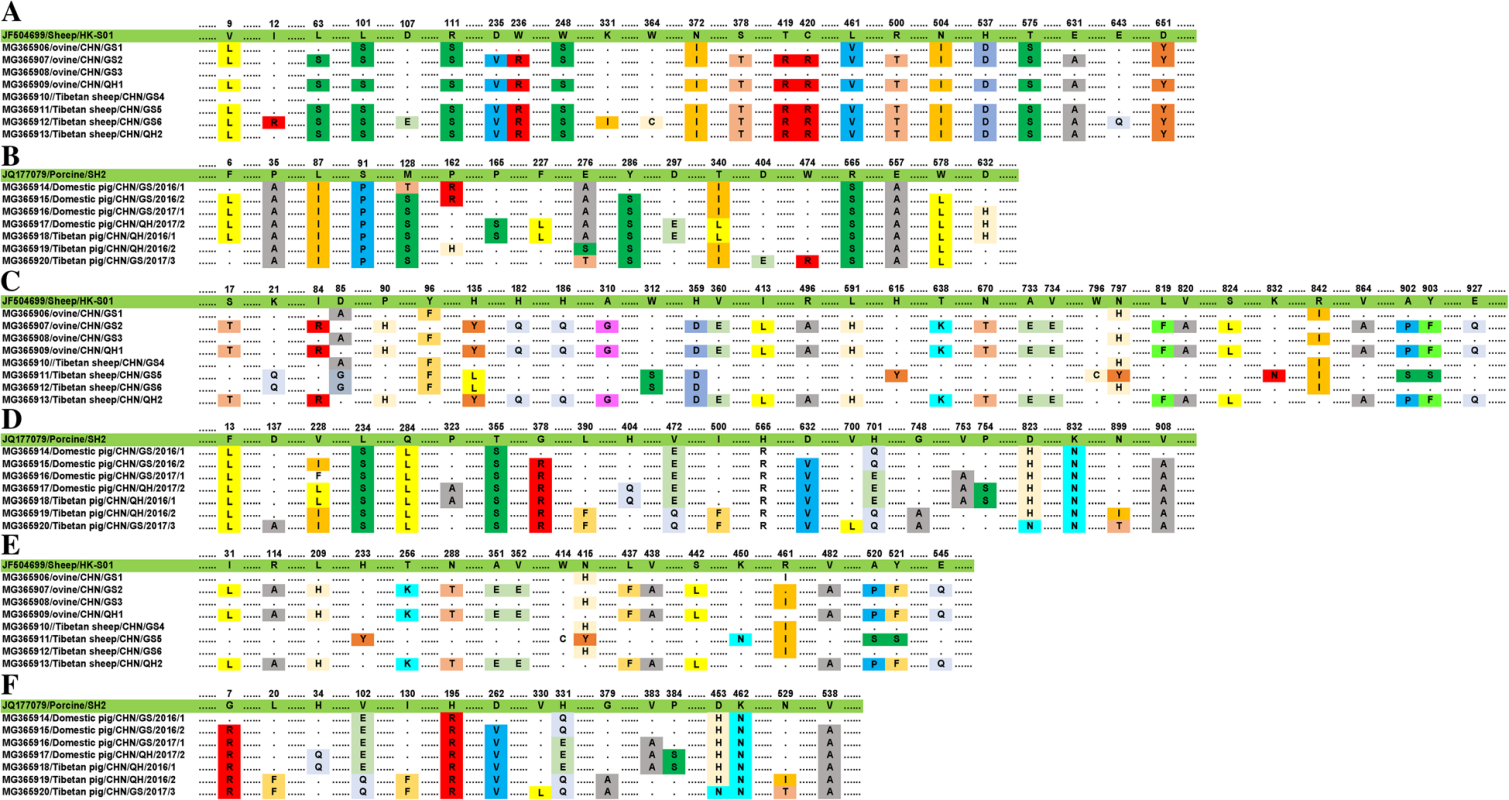
Amino acid mutation analysis of O-PARV4 and P-PARV4 strains based on NS1, VP1 and VP2 sequences. A: Mutation analysis of NS1 sequences of O-PARV4 strains; B: Mutation analysis of NS1 sequences of P-PARV4 strains; C: Mutation analysis of VP1 sequences of O-PARV4 strains; D: Mutation analysis of VP1 sequences of P-PARV4 strains; E: Mutation analysis of VP2 sequences of O-PARV4 strains; F: Mutation analysis of VP2 sequences of P-PARV4 strains

To identify the amino acid changes in the NS1, VP1 and VP2 proteins of the O-PARV4 and P-PARV4 strains in this study, we aligned the deduced amino acid sequences of the full-length NS1, VP1 and VP2 sequences with JF504699/Sheep/HK-S01 and JQ177079/Porcine/SH2, respectively. As shown in Fig. 3, the mutation could be observed in the NS1, VP1 and VP2 sequences of most O-PARV4 and P-PARV4 strains. However, there were no amino acid substitutions in the NS1 sequences of the two strains of O-PARV4 (MG365908/ovine/CHN/GS3 and MG365910/Tibetan sheep/CHN/GS4), and the numbers of mutations were also lower in the VP1 and VP2 sequences. Furthermore, the number of mutations of deduced amino acids in the NS1, VP1 and VP2 sequences of O-PARV4 was higher than that in P-PARV4, and no recombination was found in these strains (Fig. 3).

## Discussion

The comprehensive investigation in our present study demonstrated a widespread distribution of PARV4-related viruses in livestock in the provinces surrounding the Qinghai-Tibet Plateau in China. All these data indicated that the prevalence of P-PARV4 and O-PARV4 differed among the different animals, ages and areas, which is consistent with the results of the PARV4 prevalence in domestic animals in other countries and the provinces in China [4, 5, 14–16]. Therefore, detection and further analysis of PARV4-related viruses from different geographic areas will be helpful for understanding the worldwide distribution and heterogeneity of PARV4-related viruses in animals and their potential zoonotic potential.

Similar to yaks, Tibetan pigs and Tibetan sheep have evolved over thousands of years as one of the unique and indigenous breeds in China. By living in cold and harsh environments on plateaus for a long time, the special livestock on the Qinghai-Tibet Plateau have undergone specific selection to enrich disease resistance-related gene categories in their genome [6, 7, 17, 18]. The results of this study showed a lower prevalence of P-PARV4 in Tibetan pigs and O-PARV4 in Tibetan sheep than in domestic pigs and ovines. The same phenomenon was also observed for B-PARV4 infections in yaks [3]. The cold and harsh environment might be another factor that affects the transmission and infection of pathogens and results in a lower prevalence of PARV4-related viruses in livestock on the Qinghai-Tibet Plateau. Additionally, other pathogens infecting Tibetan pigs and yaks also provide information that the special livestock on the Qinghai-Tibet Plateau show striking physiological differences from lowland piglets, such as HEV infection in yak [3] and SIV and HP-PRRSV in Tibetan pigs [11, 12]. Both of these studies show the effects of the environment on pathogen transmission and infection, and these factors should not be ignored in future studies, especially on the Qinghai-Tibet Plateau.

The most interesting data in this study were from the molecular characterization of PARV4-related viruses in livestock on the Qinghai-Tibet Plateau, which was confirmed by phylogenetic and identity analyses using 13 novel and 30 previously reported PARV4-related whole-genome sequences (Fig. 1), which were consistent with those of phylogenetic analyses based on full-length NS1, VP1, and VP2 sequences obtained by the maximum likelihood (ML) method (Fig. 2). Phylogenetic analysis showed that all of the current P-PARV4 strains in this study are located on one branch and are closely related to strain JQ177079/Porcine/SH2 discovered in Shanghai in eastern China [4], suggesting that they might have evolved from the same ancestors. For the O-PARV4 strains in this study, there are three separate subclusters in the phylogenetic tree based on whole-genome sequences of PARV4-related strains. A distant relation was also shown by a phylogenetic tree based on full-length NS1, VP1, and VP2 sequences for MG365908/ovine/CHN/GS3 and MG365910/Tibetan sheep/CHN/GS4. The nucleotide and amino acid sequence identities of NS1 in these two O-PARV4 strains to JF504699/Sheep/HK-S01 were 100% (Fig. 3), and four and two amino acid mutations were found in VP1 and VP2 sequences of these two O-PARV4 strains, respectively. These results demonstrated that the O-PARV4 strain in Tibetan sheep and ovines was the same strain as JF504699/Sheep/HK-S01 and was transmitted from Hong Kong, which needs further study. In addition, the high number of amino acid changes in O-PARV4 strains indicated that the mutation possibility of O-PARV4 is higher than that in P-PARV4, and more studies are needed to confirm this finding.

## Conclusions

Collectively, our study is the first to demonstrate the presence of P-PARV4 in Tibetan pigs and O-PARV4 in Tibetan sheep, which provides a better understanding of the molecular epidemiology, genetic diversity and phylogenetic relationships of PARV4-related viruses in livestock in China and other PARV4-related reference strains. Until now, no evidence of cross-species infection or transmission has been reported, and the detection and further analysis of PARV4-related viruses in livestock and other animals on the Qinghai-Tibet Plateau will help to clarify the source, circulation pattern, zoonotic potential and public health risk of this virus. The mechanisms of its pathogenicity, transmission, evolution and persistence also require urgent clarification.

## Additional files

Additional file 1: Detection rates of ungulate tetraparvovirus 2 (P-PARV4) in domestic pigs and Tibetan pigs, ungulate tetraparvovirus 4 (O-PARV4) in ovine and Tibetan sheep on the Qinghai-Tibetan Plateau, China. (PDF 102 kb)

Additional file 2: Primers used for detection and full-length genome amplification of P-PARV4 and O-PARV4 in different hosts. (PDF 102 kb)

## Abbreviations

B-PARV4: Ungulate tetraparvovirus 1
BVDV: Bovine viral diarrhoea virus
HEV: Hepatitis E virus
HP-PRRSV: Highly pathogenic porcine reproductive and respiratory syndrome virus
ICTV: International Committee on Taxonomy of Viruses
NS: Non-structural proteins
O-PARV4: Ungulate tetraparvovirus 4
ORFs: Open reading frames
P-PARV4: Ungulate tetraparvovirus 2
SIV: Swine influenza A virus
VP: Viral capsid proteins
