# Supplementary material for: First detection and genetic characterization of ungulate tetraparvovirus 2 and ungulate tetraparvovirus 4 in special livestock on the Qinghai-Tibet Plateau in China
Source: Virol J. 2019 May 2;16:56. doi: 10.1186/s12985-019-1167-z (PMC6498466; doi:10.1186/s12985-019-1167-z)
Supplement: Supplementary file 1 — Detection rates of ungulate tetraparvovirus 2 (P-PARV4) in domestic pigs and Tibetan pigs, ungulate tetraparvovirus 4 (O-PARV4) in ovine and Tibetan sheep on the Qinghai-Tibetan Plateau, China. (PDF 102 kb) [file 12985_2019_1167_MOESM1_ESM.pdf]

Additional file 1. Detection rates of ungulate tetraparvovirus 2 (P-PARV4) in domestic pigs and Tibetan pigs, ungulate tetraparvovirus 4 (O-PARV4) in ovine and Tibetan sheep on the Qinghai-Tibetan Plateau, China.

Species (Animals)	Location (Province)	Age (Months)	No[a]	P-PARV4 (%)[b]	P-PARV4 in all samples (%)[c]	O-PARV4 (%)[b]	O-PARV4 in all samples (%)[c]
Domestic pigs	Gansu	≤ 1	64	13/64(20.31)	22/116(18.97)		
		> 1	52	9/52(17.31)			
	Qinghai	≤ 1	59	8/59(13.56)	12/102(11.76)		
		> 1	43	4/43(9.30)			
Tibetan pigs	Gansu	≤ 1	49	8/49(16.33)	13/91(14.29)		
		> 1	42	5/42(11.90)			
	Qinghai	≤ 1	38	2/38(5.26)	4/90(4.44)		
		> 1	45	2/52(3.85)			
Ovine	Gansu	≤ 1	67			5/67(7.46)	8/121(6.61)
		> 1	54			3/54(5.56)	
	Qinghai	≤ 1	72			6/72(8.33)	10/125(8.00)
		> 1	53			4/53(7.55)	
Tibetan sheep	Gansu	≤ 1	62			4/62(6.45)	5/110(4.55)
		> 1	48			1/48(2.08)	
	Qinghai	≤ 1	45			3/45(6.67)	6/109(5.50)
		> 1	63			3/63(4.76)	

[a] The number of domestic pigs, tibetan pigs, ovine, tibetan sheep samples collected from each provinces with two age groups in the study.

[b] Number and percentage of positive samples in each age group of different provinces.

[c] Number and percentage of positive samples in all age groups of different provinces.
