# Supplementary material for: First detection and genetic characterization of ungulate tetraparvovirus 2 and ungulate tetraparvovirus 4 in special livestock on the Qinghai-Tibet Plateau in China
Source: Virol J. 2019 May 2;16:56. doi: 10.1186/s12985-019-1167-z (PMC6498466; doi:10.1186/s12985-019-1167-z)
Supplement: Supplementary file 2 — Primers used for detection and full-length genome amplification of P-PARV4 and O-PARV4 in different hosts. (PDF 102 kb) [file 12985_2019_1167_MOESM2_ESM.pdf]

Additional file 2 Primers used for detection and full-length genome amplification of P-PARV4 and O-PARV4 in different hosts.

Primers	Sequences	Binding position[a]	Length[b]	Function[c]	Reference
PARV4-F	5'-GGACYTCTATGYTRGCTGAYCG-3'	3404-4043(EU200676)	640	PARV4 detection in domestic pigs,	EU200676 and
PARV4-R	5'-TKATCKACMCCWGTCATGATMGC-3'	3518-4157(JF504700)		Tibetan pigs, ovine and Tibetan sheep.	JF504700
P-PAPV4-F1	5'-CCTATAAGAATCAGTGTCAGTTCC-3'	64-1529	1466	Amplify full-length P-PARV4 genomes	EU200676
P-PAPV4-R1	5'-CTCAGATCTCTTTCTGGCAACG-3'			in domestic pigs and Tibetan pigs	
P-PAPV4-F2	5'-CCTACAGACAAGAATAACTATG-3'	1449-3304	1856		
P-PAPV4-R2	5'-CAGCCATGATTGTAGCAGGATCA-3'				
P-PAPV4-F3	5'-GCAGATGTTCAATTATCACAGG-3'	3127-4358	1232		
P-PAPV4-R3	5'-TCCTCTGTGTGGCCGGTATAACT-3'				
P-PAPV4-F4	5'-GTTCCTAAGTGGAAAAGGCCAG-3'	4051-5014	964		
P-PAPV4-R4	5'-GTCAGACATCTTTGTTGCATCAG-3'				
O-PAPV4-F1	5'-CACTTCCGCATTAGTGCTGAATC-3'	64-1940	1877	Amplify full-length O-PARV4 genomes	JF504700
O-PAPV4-R1	5'-GAGGTACCTTTGATATTCCTCGTC-3'			in ovine and Tibetan sheep	
O-PAPV4-F2	5'-GATAGTGAAGATGATTGGTTCC-3'	1761-2906	1146		
O-PAPV4-R2	5'-GGAGTAGTTCCTCTACCACTGTC 3'				
O-PAPV4-F3	5'-GTGCTATATGACCTGTATAGATC-3'	2689-4253	1565		
O-PAPV4-R3	5'-TCAGTGGGCATCATTAGAGATG-3'				
O-PAPV4-F4	5'-GAACCATGTTCCTGGCCCATCTC-3'	4215-5122	908		
O-PAPV4-R4	5'- CAAGAATCAGGCTCAGACACAC-3'				

F, forward; R, reverse orientation; Y = C/T; K = G/T; R = A/G; M = C/A; W = T/A;

[a] Nucleotide positions are according to the genomes of Porcine hokovirus strain HK6 (EU200676) and Ovine hokovirus strain HK-S04(JF504700).

[b] Amplicon size is given in base pairs

[c] The aim of the primers used in this study.
